# Natural Cytotoxicity Receptors: Broader Expression Patterns and Functions in Innate and Adaptive Immune Cells

**DOI:** 10.3389/fimmu.2013.00069

**Published:** 2013-03-20

**Authors:** Kelly Hudspeth, Bruno Silva-Santos, Domenico Mavilio

**Affiliations:** ^1^Unit of Clinical and Experimental Immunology, Humanitas Clinical and Research CenterRozzano, Milan, Italy; ^2^Department of Medical Biotechnologies and Translational Medicine, University of MilanMilan, Italy; ^3^Instituto de Medicina Molecular, Faculdade de Medicina, Universidade de LisboaLisboa, Portugal

**Keywords:** NCRs, T cells, activation, homeostasis, mucosal immunity

## Abstract

Natural cytotoxicity receptors (NCRs) have been classically defined as activating receptors delivering potent signals to Natural Killer (NK) cells in order to lyze harmful cells and to produce inflammatory cytokines. Indeed, the elicitation of NK cell effector functions after engagement of NCRs with their ligands on tumor or virus infected cells without the need for prior antigen recognition is one of the main mechanisms that allow a rapid clearance of target cells. The three known NCRs, NKp46, NKp44, and NKp30, comprise a family of germ-line encoded Ig-like trans-membrane (TM) receptors. Until recently, NCRs were thought to be NK cell specific surface molecules, thus making it possible to easily distinguish NK cells from phenotypically similar cell types. Moreover, it has also been found that the surface expression of NKp46 is conserved on NK cells across mammalian species. This discovery allowed for the use of NKp46 as a reliable marker to identify NK cells in different animal models, a comparison that was not possible before due to the lack of a common and comprehensive receptor repertoire between different species. However, several studies over the recent few years indicated that NCR expression is not exclusively confined to NK cells, but is also present on populations of T as well as of NK-like lymphocytes. These insights raised the hypothesis that the induced expression of NCRs on certain T cell subsets is governed by defined mechanisms involving the engagement of the T cell receptor (TCR) and the action of pro-inflammatory cytokines. In turn, the acquisition of NCRs by T cell subsets is also associated with a functional independence of these Ig-like TM receptors from TCR signaling. Here, we review these novel findings with respect to NCR-mediated functions of NK cells and we also discuss the functional consequences of NCR expression on non-NK cells, with a particular focus on the T cell compartment.

## Introduction

Natural Killer cell Receptors (NKRs) comprise several trans-membrane (TM) inhibitory and activating molecules that regulate natural killer (NK) cell function and homeostasis. Within this group are Killer-cell-Ig-like-receptor (KIR), lectin type receptors as well as Natural Cytotoxicity Receptors (NCRs). The ability of NK cells to perform effector functions is determined by a delicate balance of signals received from these receptors. Activating NKRs, including NCRs, are germ-line encoded proteins, which allow NK cells, differently from T cells, to respond to harmful cells without prior antigen sensitization (Vivier et al., 2011). Although most of the NKRs were originally identified on NK cells, they have also been reported as constitutively expressed by some T lymphocyte populations (Raulet et al., 2001; Lanier, 2005). Nonetheless, it was thought that NCRs, and in particular NKp46 and NKp30, were present exclusively on resting NK cells (Pessino et al., 1998; Pende et al., 1999; Moretta et al., 2002). Recent data has revealed, however, that NCRs are expressed on other populations of cells, such as T cells and NK-like cells (Meresse et al., 2006; von Lilienfeld-Toal et al., 2006; Stewart et al., 2007; Walzer et al., 2007b; Tang et al., 2008; Bensussan et al., 2011; Correia et al., 2011; Hudspeth et al., 2012). In particular, the functions of NCRs on certain T cell subsets appears to mirror the ones of the NCRs expressed on NK cells. In this regard, the main working hypothesis is that T lymphocytes employ these activating receptors to circumvent antigen-restricted responses, thus allowing them to respond rapidly against dangers to the host. Alternatively, it has also been speculated that the expression of NCRs may represent a marker for those T cells that have undergone chronic activation and have lost immune tolerance (Meresse et al., 2006; Stewart et al., 2007; Correia et al., 2011). Here, we review the recent findings regarding the phenotypic distribution of NCRs on non-NK cell types and their functional consequences on immune responses.

## Identification and Molecular Structure of NCRs

The identification and cloning of three NCRs, NKp46, NKp30, and NKp44, was achieved in the late 1990s and revealed that NKp46 and NKp30 are expressed by NK cells freshly purified from peripheral blood, whereas NKp44 is induced upon NK cell activation (Sivori et al., 1997; Pessino et al., 1998; Vitale et al., 1998; Cantoni et al., 1999).

**Figure 1:**
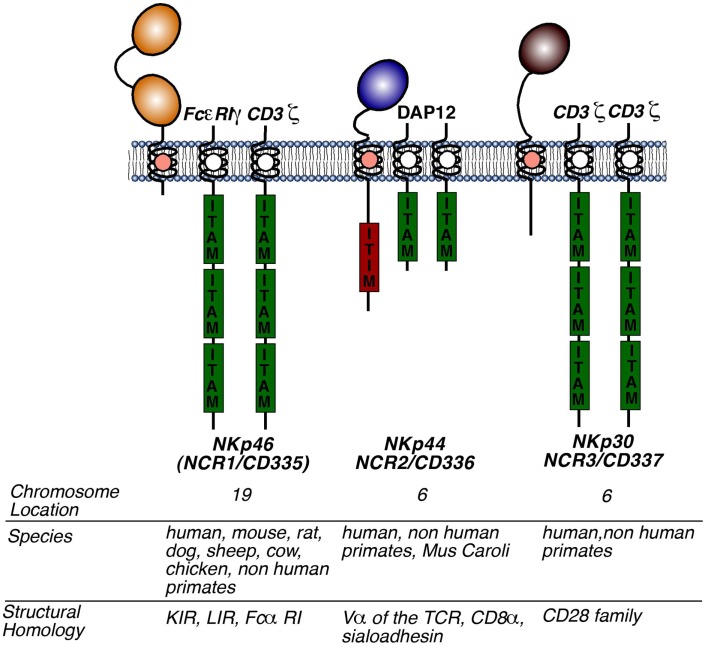
**Structure of NCRs**. Molecular structures of NKp46, NKp30, and NKp44 including the presence and the number of extracellular domains, the type of adaptor molecules (to the right to each NCR), the chromosomal location, the species in which a functional gene is present, and family of receptors homologous to the respective NCR. Abbreviations: KIR, killer immunoglobulin receptor; LIR, leukocyte immunoglobulin-like receptor; ITAM, immunoreceptor tyrosine-based activation motif; ITIM, immunoreceptor tyrosine-based inhibition motif.

Natural cytotoxicity receptors are type I TM receptors that, unlike T cell receptors (TCRs) and immunoglobulins, do not undergo any somatic recombination [i.e., V(D)J genetic rearrangements] in order to become functionally active. Moreover, the characterization of NCRs using crystallography has revealed that they are structurally quite different from each other, despite belonging to the same group of receptors and performing similar functions (Cantoni et al., 2003; Foster et al., 2003; Li et al., 2011). The NKp46 gene (*NCR1*) encodes for an Ig-like receptor, which contains two extracellular Ig domains, a type I TM domain and a short cytoplasmic tail. Unlike NCR1, the genes encoding for NKp30 and NKp44, *NCR2* and *NCR3*, give rise to type I TM proteins with only one extracellular Ig domain. The TM regions of all three NCRs are coupled with adaptor molecules given their lack of inherent signaling motifs (Figure 1). Following the ligation of NCRs with their putative ligands, these adaptor molecules allow for intracellular activating signaling through immune-receptor tyrosine-based activating motifs (ITAMs). Notably, the cytoplasmic domain of NKp44 contains an immunoreceptor tyrosine-based inhibitory (ITIM) motif that, in contrast to ITAM motifs, impart negative signals and are contained within inhibitory receptors such as KIRs (Pessino et al., 1998; Vitale et al., 1998; Cantoni et al., 1999; Pende et al., 1999; Campbell et al., 2004).

## NCR Functional Correlates on NK Cells

### NCR-mediated elimination of tumor-transformed cells

Natural cytotoxicity receptors were originally identified as NKRs that have the ability to mediate the killing of tumor-transformed cells (Sivori et al., 1997; Pessino et al., 1998; Vitale et al., 1998; Pende et al., 1999; Moretta et al., 2001). Indeed, it soon became clear that NCRs are able to arm NK cells with the ability to respond robustly and with high efficiency against tumor-transformed cells. While the killing of certain tumor cells by NK cells sometimes involves more than one NKR, the lysis of other tumor cells can be entirely mediated by NKp46 or NKp30 or NKp44, thus highlighting the unique ability of each one of the three NCRs to individually trigger NK cell cytotoxicity (Moretta et al., 2001). Other factors determining NK cell-mediated lysis of tumor cells are the presence of NCR ligands on the surface of targets (Byrd et al., 2007; Halfteck et al., 2009) as well as the surface levels of NCRs on NK cell surface (Sivori et al., 1999). In fact, NKp44 is expressed only on activated circulating NK cells both *in vitro* and *in vivo*, while the stimulation of NK cells with cytokine such as IL-2 increases the constitutive expression and the cytolytic potential of NKp46 and NKp44 (Moretta et al., 2001; Mavilio et al., 2003). In addition and confirming the key role of NK cells in the clearance of certain tumors *in vivo* (Smyth et al., 2002), several studies have demonstrated that the absence of NKp46 results in an impaired eradication of certain tumors, such as lymphoma and melanoma (Gazit et al., 2006; Halfteck et al., 2009; Lakshmikanth et al., 2009; Glasner et al., 2012).

### NCR-mediated clearance of cells infected by pathogens

Along with their ability to eliminate tumor-transformed cells, NCRs have also been implicated in the control and elimination of several pathogens. In fact, NKp46 has been shown to be required for the eradication of bacteria and virus infection *in vivo*, since NK cells were unable to recognize and eliminate infected cells expressing NKp46 ligands in NKp46-deficient mice inoculated with influenza virus (Gazit et al., 2006). Another representative example of the central role of NCRs in the control of virus infection was shown in an *in vitro* model of human cytomegalovirus (HCMV) infection (Magri et al., 2011). In the present study, authors demonstrated that the clearance of HCMV-infected monocyte derived dendritic cells (MDDCs) is associated with the down-modulation of self major histocompatibility complex of class I (MHC-I) molecules, whose interactions with inhibitory NK cell receptors (iNKRs) normally switch off NK cell effector functions. The lack or decreased engagement of iNKRs with their putative self-MHC-I ligands makes it possible for NK cells to recognize and kill harmful HCMV-infected MDCCs through the direct recognition of a self-encoded NKp46 ligand on these target cells (missing self hypothesis) (Ljunggren and Karre, 1990).

Similarly, NKp46 has also been demonstrated to play a key role in the recognition and clearance of *Streptococcus pneumoniae*. Indeed, NKp46-deficient mice were reported to have reduced NK cell activation and interferon-γ (IFN-γ) production during the course of early *S. pneumoniae* infection in the lungs. In contrast, NKp46-expressing wild type mice appear to be endowed with potent alveolar macrophage responses as compared to NCR1-deficient mice. This result correlates with the higher fraction of NKp46 ligand on lung macrophages in NCR1-expressing mice that are also equipped with better phagocytic activity compared to that of macrophages with lower or negative surface levels of NKp46 ligands (Elhaik-Goldman et al., 2011).

Natural cytotoxicity receptors have also been shown to play an important role in the pathogenesis of HIV-1 infection. First, our group identified a pathologic expansion of a subset showing an abnormal receptor repertoire that greatly impairs NK cell cytolytic and immune-regulatory functions (Fauci et al., 2005; Brunetta et al., 2010). In particular, the expression of NKp46 and NKp30 is remarkably reduced on circulating and freshly purified NK cells from HIV-1 infected patients with high levels of chronic viremia, and this is directly associated with the decreased ability of NK cells to lyze NCR-ligand-positive tumor cell lines (De Maria et al., 2003; Mavilio et al., 2003, 2005). In addition to the impairment in NK cell function, it is well known that HIV-1 viremia induces a CD4^pos^ T cell depletion that leads to immunodeficiency and correlates with disease progression. However, it has also been reported that the disappearance of the majority of CD4^pos^ T cells during infection are not productively infected with HIV-1 (Alimonti et al., 2003). One possible explanation is that these uninfected CD4^pos^ T cells are eliminated through a mechanism not directly linked to viral replication. In this regard, our group demonstrated both *in vitro* (Ward et al., 2007) and *ex vivo* (Fogli et al., 2008) that HIV-1 replication can induce the expression of ligands for NKp46, NKp30, and NKp44 on uninfected CD4^pos^ T cells. The fact the HIV-1 is able to specifically induce the expression of NCR ligands on uninfected cells has also been confirmed by another study demonstrating that a highly conserved motif of HIV-1 gp41 envelope protein can induce the expression of NKp44 ligand on uninfected CD4^pos^ T cell blasts and render these cells susceptible to NK cell-mediated killing via NKp44 (Vieillard et al., 2005). Indeed, in a model of simian human immunodeficiency virus (SHIV) infected non-human primates, immunization with the 3S gp41 peptide has been shown to prevent the expression of NKp44 ligand on CD4^pos^ T cells, thus lowering the depletion of these cells (Vieillard et al., 2008).

On the other hand and according to the aforementioned “missing self hypothesis” (Ljunggren and Karre, 1990), NK cells should be able to recognize and kill CD4^pos^ HIV-1 infected cells, which have been shown to selectively down-modulate self human leukocyte antigens allele A and B (HLA-A and -B) (Ward et al., 2007; Fogli et al., 2008). In this regard, we have to also take into account that HLA-C and HLA-E alleles are not down-regulated on CD4^pos^ T cells upon infection with HIV-1 and this would protect, at least in part, HIV-1 infected cells from NK cell killing. However, not all circulating NK cells express specific inhibitory receptors recognizing HLA-C and -E. In particular, only a fraction of the main cytotoxic CD56^dim^/CD16^pos^ NK cells express NKG2A or LIR1/ILT2. Therefore, other mechanisms are required to explain the impaired NK cell cytolytic activity against autologous, endogenously HIV-1 infected CD4^pos^ T cells. We have shown that this phenomenon is also due to the defective surface expression and function of NCRs, while the residual NK cell-mediated killing of HIV-1 infected cells occurs mainly through NKG2D, the only main activating NK cell receptor whose surface expression is not affected by high levels of plasma viremia (Mavilio et al., 2003, 2005) and whose ligands on HIV-1 infected cells have been demonstrated to be induced by ongoing viral replication (Ward et al., 2007; Fogli et al., 2008). All of the above-mentioned experimental evidence highlight the key contribution of NCRs to the physiopathology of HIV-1 infection, since their impaired functions greatly affect the clearance of the virus and contribute to the related acquired immune-deficiency and progression to AIDS.

### NCR-mediated regulation of immune homeostasis

It has recently become clear that the function of NCRs is not only in the induction of NK cell lysis of harmful tumor-transformed or infected cells, but they also play a major role in regulating the homeostasis of immune responses. In the context of the recently disclosed cross-talk between NK cells and autologous dendritic cells (DCs), NKp30 is able to edit the maturation of DCs by killing unresponsive, aberrant, or immature DCs (iDCs) and by sparing properly matured DCs (mDCs) that can then migrate to the secondary lymphoid organs. The final outcome of this NKp30-mediated interaction is the coordination and optimization of a correct DC priming in order to develop an antigen-specific T cell response. The engagement of NKp30 on NK cells in the context of their interaction with autologous DCs highly contributes to establish important links between innate and adaptive immune responses through a process that requires both NK cell-DC cellular contacts and secretion of specific cytokines (Ferlazzo et al., 2002; Gerosa et al., 2002; Moretta, 2002; Cooper et al., 2004; Raulet, 2004; Degli-Esposti and Smyth, 2005; Walzer et al., 2005). Our group reported that NK cell-DC interactions are markedly impaired and partially disrupted during the course of chronic and active HIV infection due to the decreased expression and impaired function of NKp30 on NK cells. This defect leads to an abnormal maturation of DCs that, upon migration to secondary lymphoid organs, are defective in priming an optimal HIV-1 specific T cell immune response and also contribute to spread the infection (Mavilio et al., 2006; Brunetta et al., 2010).

Natural cytotoxicity receptors have also been shown to be involved in the NK cell killing of polymorphonuclear neutrophils expressing NKp46 ligand. Indeed, co-culture experiments revealed that human NK cells could trigger caspase-dependent neutrophil apoptosis in a cell contact-dependent manner, which is mediated by NKp46 as well as the Fas/Fas ligand pathway (Thoren et al., 2012). To further support their findings *in vitro*, the authors used an *in vivo* human model of neutrophil extravasation where NKp46^pos^ NK cells were seen to infiltrate areas of cutaneous inflammation, populated by neutrophils. Additionally, the authors noted a positive correlation between the appearance of NKp46^pos^ NK cells and neutrophil apoptosis. This study provides evidence that apoptosis mediated by NKp46 on NK cells assists in the termination of inflammation by eliminating effector cells to avoid an unwanted immune response.

As described above, NCRs have long been assumed to act solely as activating receptors for NK cells and to serve as a key component in the innate arm of the immune response. However, in a recent report NKp46 was ascribed to have quite a different immune-regulatory function on NK cells (Narni-Mancinelli et al., 2012). This was demonstrated in a mouse model (Noè mice) bearing an NKp46 loss-of-function mutation, which resulted in a hyper-responsive NK cell response and in an increased resistance to murine CMV (MCMV) and influenza virus infections. The increased NK cell activity was associated with a decreased ability to prime T cells during infection, which resulted in a diminished memory T cell response after antigen challenge. Interestingly, expression of the transcript levels for *Helios*, a member of the Ikaros transcription factor family genes, inversely correlated with the presence of NKp46. Reintroducing NKp46 into the loss-of-function mouse strain restored mRNA levels of *Helios* and was critical for the subsequent development of optimal anti-viral and antibacterial T cell responses. Taken together, these data suggest that NKp46, other then being an activating receptor triggering NK cell cytotoxicity, is also endowed with regulatory functions which ultimately lead to a correct priming of antigen-specific adaptive immune responses against virus infections. While these results are certainly innovative and disclose an unpredicted NKp46-mediated NK cell functional correlate, we have to take into account, as also stated by the same authors, that this NCR is constitutively present on the surface of a CD3^pos^ T cell subset in the spleen of Noè mice and that the NKp46 mutation significantly affects its expression on this T cell population as well.

### NCR-mediated production of pro-inflammatory cytokines

In addition to their ability to induce cytotoxicity, NCRs can also mediate the production of pro-inflammatory cytokines by NK cells. Early experiments showed that cross-linking of NKp46 and NKp44 resulted in the production of IFN-γ and tumor necrosis factor-α (TNF-a) (Sivori et al., 1997; Vitale et al., 1998). It is well known that the production of these pro-inflammatory cytokines is essential for the clearance of bacterial and viral pathogens as well as in the modulation of immune responses (Pfeffer, 2003; Schroder et al., 2004). Among the several examples of the functional consequence of NCR-mediated cytokine production, there is the ability of NKp30 expressed on NK cells to bind its ligand on the surface of iDCs. This cellular interaction leads to the NK cell production of several cytokines such as IFN-γ and TNF-α which, in turn, trigger autologous DC maturation (Moretta, 2002). In particular, it has been shown that different isoforms of NKp30 are able to elicit the production of different cytokines by NK cells in response to interactions with autologous iDCs (Delahaye et al., 2011). Isoforms of NKp30 can arise due to mutations in exon 4, which results in the production of three splice variants of NKp30: the isoforms a, b, and c containing distinct intracellular domains. While the binding of NKp30a and NKp30b with their putative ligands on iDCs results in the production of high amounts of IFN-γ and TNF-α, the NKp30c isoform does not induce the NK cell synthesis of pro-inflammatory cytokines. Instead, NKp30c interaction with its ligands on iDCs results in the production of the anti-inflammatory cytokine IL-10. Moreover, the engagement of NKp30c, unlike NKp30a and NKp30b splice variants, is also associated with a reduced ability of NK cells to produce IFN-γ and to kill target cells. In line with this, it has also been demonstrated that the cross-linking of these three different isoforms induces distinct signals in NK cells since NKp30a and NKp30b, but not NKp30c, are coupled with CD3ζ, the ITAM-bearing adaptor protein required for activating downstream signaling. The correct functions of NKp30 isoforms within NK cell-DC interactions contributes to regulate the optimal maturation of DCs in the context of the NK cell-mediated editing of these antigen presenting cells (APCs). In fact, only those DCs that fail to undergo a correct maturation are then recognized by NK cells and killed via activating isoforms of NKp30 (Delahaye et al., 2011). Although several splice variants of NKp46 and NKp44 have been reported, a functional characterization of these NCR isoform has not yet been performed. It will be of interest to determine whether variants of NKp46 and NKp44 are expressed on primary human lymphocytes and if such isoforms, similarly to what has been observed for NKp30c, have different effects on NK cell functions.

Finally, the binding of NKp46 to its ligand expressed on the surface of cells infected with HCMV and *Fusobacterium nucleatum* has also been shown to trigger the production of inflammatory cytokines (Magri et al., 2011; Chaushu et al., 2012).

### Other functions of NCRs

An abundant presence of NCRs has been recently described in the human decidua where they are known to play an important and unexpected role during pregnancy. Indeed, the human uterine mucosa during pregnancy is comprised of an unusually high frequency of NK cells (up to 40% of total immune cells) (Hanna et al., 2006). Different from circulating NK cells (Moretta et al., 2001), decidual NK cells constitutively express NKp44 and this is likely due to their constant exposure in the decidua with activating receptors and pro-inflammatory cytokines. Indeed, NK cells from the decidua express high amounts of the early activation marker, CD69 (Michael and Lotze, 2010). The function of NCRs on NK cells from the decidua is unique, since their interactions with putative ligands on the surface of trophoblasts induces the synthesis by NK cells of the angiogenic factors vascular endothelial growth factor (VEGF), placental growth factor (PLGF), chemokines interferon-inducible protein-10 (IP-10), stromal cell-derived factor-1 (SDF-1), and IL-8. In turn, the NCR-mediated secretion of these proteins is able to regulate trophoblast invasion during pregnancy and induce angiogenesis in the decidua (Hanna et al., 2006; Vacca et al., 2008).

In contrast to the regulatory and “peaceful” role of NCRs in ensuring a correct homeostasis of human female genital tract during pregnancy, it has also been demonstrated that NCRs are involved in the physiopathology of autoimmunity. In fact, the induced expression of an NKp46 ligand by pancreatic β cells correlates with high levels of NKp46^pos^ NK cells infiltrating the pancreatic islets during the course of type 1 diabetes. The binding between NKp46 and its ligands has been shown to induce a remarkable NK cell degranulation that, in turn, contributes to the destruction of pancreatic islets. The important role that NKp46 plays an in the pathogenesis of autoimmune diabetes is also confirmed by the fact that the NK cell-mediated depletion of pancreatic islets is greatly reduced if not abrogated in NKp46-deficient mice or after injecting soluble NKp46 in wild type mice (Gur et al., 2010).

Natural cytotoxicity receptors have also been shown to be involved in the pathogenesis of periodontitis, a condition caused by *F. nucleatum* and *Porphyromonas gingivalis*, which results in tooth loss. Although it is clear that these bacteria initiate periodontitis, the progression of the disease is actually caused by the hosts’ innate and adaptive immune systems through the production of inflammatory cytokines. In this context, it has been reported that NCRs play a role in the exacerbation of periodontal disease in a mouse model of periodontitis, since *F. nucleatum* is able to directly bind NKp46 on NK cells that, in turn, produce high levels of TNF-α (Chaushu et al., 2012).

## Regulation of NCR Expression

Once it became evident that NCRs harness a remarkable potential as targets in the development of therapies for the clearance of tumor-transformed cells, researchers became interested in developing methodological approaches to implement and optimize the expression of NCRs on NK cells. As pointed out earlier, NKp46 and NKp30 are constitutively expressed on NK cells, whereas NKp44 is induced upon NK cell activation with IL-2 (Cantoni et al., 1999; Moretta et al., 2001). It has been subsequently shown that surface expression of NKp44 is also increased using other γc cytokines such as IL-15 (de Rham et al., 2007). Interestingly, IL-21 can prevent the induction of NKp44 by either IL-2 or IL-15 due to its ability to down-modulate DAP-12, an adaptor molecule required for signaling and stable surface expression of NKp44 (Campbell et al., 2004; de Rham et al., 2007). In contrast, NCRs are down-modulated *in vitro* by TGF-β, but not after incubation with either IL-10 or IL-4, which suggests that anti-inflammatory cytokines may have an important role in the suppression of NCR-mediated NK cell responses (Castriconi et al., 2003). Moreover, a decrease in the surface expression of NCRs has also been detected on NK cells from patients with chronic immune activation occurring during several diseases such as HIV-1 infection and tumors (Costello et al., 2002; De Maria et al., 2003; Mavilio et al., 2003; Fauriat et al., 2007; Garcia-Iglesias et al., 2009). The differential modulation of NKp46, NKp30, and NKp44 in various settings both *in vitro* and *in vivo* implies that NCRs are not entirely redundant and can be specifically modulated to eliminate unwanted and dangerous cellular targets in the context of distinct immune responses.

In contrast to the vast amount of data available regarding the cell surface modulation of NCRs, there is a relative paucity of information with respects to their transcriptional regulation. Some insight has, however, been provided by the investigation of the mechanisms of NKp46 expression regulation. Extensive examination of the presence of mRNA for NCR1 in various mouse and human cells has revealed that this gene is constitutively expressed in NK cells, which suggests the existence of a transcriptional control of NKp46 expression (Pessino et al., 1998; Lai and Mager, 2012). Indeed, two regions upstream of the NCR1 gene have been identified in mice and humans. They contain promoter activities and control the expression of NKp46 on NK cells (Walzer et al., 2007a; Lai and Mager, 2012). It was shown that one of these regions contains an essential promoter, which includes a runt-related transcription factor (RUNX) motif. The second region was identified as a promoter enhancer with a RUNX and an ETS binding motif. Truncation mutants revealed that the essential promoter confers global expression of NCR1, while the region containing the enhancer was found to have either enhancer activity or suppressor activity depending on the cell type. Indeed, this enhancer region augmented the essential promoter activity when expressed in NK cells, while it suppressed promoter activity when expressed in cells other than NK lymphocytes. The regulation of the enhancer in regard to NKp46 gene expression in NK cells is dependent on RUNX3, whereas it has no effect on the essential promoter. While the promoter element also contains an ETS binding motif, its ability to regulate NKp46 expression has not yet been disclosed.

## Ligands for NCRs

Over the past decade, intensive and important work has been performed in order to elucidate the identity of NCR ligands. Unlike other NK cell receptors, which in general bind self-MHC and MHC-related proteins, NCRs appear to recognize a different set of ligands that include pathogen-derived molecules as well as non-MHC self-molecules expressed on stressed cells.

### Pathogen components as NCR ligands

Several reports have described that the hemagglutinin (HA) protein of the influenza and vaccinia virus can bind NKp46 and stimulate NK cells to lyse virus infected cells (Mandelboim et al., 2001; Jarahian et al., 2011). While vaccinia virus HA can act to stimulate NK cell activity through NKp46, this viral glycoprotein was also shown to inhibit NK cell function through the binding of NKp30. Additionally, the recognition of Sendai and Newcastle viruses can also occur through NKp44 and NKp46 (Mandelboim et al., 2001; Arnon et al., 2005; Jarahian et al., 2011).

Natural killer cell activation by pathogens via NCRs appears not only to be limited to viruses but it has also been reported for intracellular bacteria and parasites. The Duffy binding-like 1α domain within the *Plasmodium falciparum* erythrocyte binding protein of the malaria parasite was shown to bind and activate NK cells through NKp46 and NKp30 (Mavoungou et al., 2007). Moreover, NK cell recognition of *Mycobacterium tuberculosis* via the host protein vimentin expressed on the surface of infected monocytes was also reported to occur through NKp46 (Garg et al., 2006).

While many pathogens have been shown to activate NK cells through NCRs, a component of the HCMV virus, pp65, was reported to inhibit NK cell lysis of HCMV-infected cells as well as to impair NK cell-mediated killing of susceptible tumor cells and iDCs through the NKp30 receptor. The mechanism explaining the HCMV-induced inhibition of NK cell effector functions is based on the ability of pp65 to promote the dissociation of NKp30 from the CD3ζ chain, an ITAM-bearing adaptor molecule required for activating the downstream signal of NKp30 following the binding with its putative ligand (Pende et al., 1999; Arnon et al., 2005).

### Self-molecules as NCR ligands

Several studies have revealed that ligands for NCRs are not only elements from pathogens, but include self-derived molecules as well. The identification of these molecules on tumor and/or stressed cells is of vital importance to understand their interactions with NK cells and for the development of novel cancer therapies. In this regard, it has been described that several putative endogenous ligands expressed by tumor cells are capable of eliciting an NK cell response through NCRs.

One of the first self-molecules identified to interact with NKp30 is the Human Leukocyte Antigen-B-Associated Transcript 3 (BAT3) (Pogge von Strandmann et al., 2007). The expression of this molecule on tumor cell surface triggers NK cell killing and production of TNF-α and IFN-γ following the engagement of NKp30. The stimulatory ability of BAT3 has also been confirmed *in vivo* by experiments showing that peripheral blood NK cells injected into nude mice are not as efficient in clearing tumors when a blocking antibody to BAT3 was administered. It was subsequently demonstrated that even the killing of iDCs by autologous NK cells occurs through the secretion of BAT3 by iDCs in response to non-lethal heat shock. Therefore, the expression of BAT3 on iDCs plays a key role in the NK cell editing of autologous DC maturation given that the presence of this NKp30-ligand on the surface of iDCs can lead to the clearance of aberrant or improperly mDCs (Simhadri et al., 2008). Moreover, the presence of BAT3 was shown not to affect the binding of HCMV pp65 to NKp30, thus indicating that NKp30 contains more than one epitope for the recognition of multiple ligands.

Another important study identified a new member of the B7 receptor family, B7–H6, as another ligand for NKp30 (Brandt et al., 2009). B7–H6 is present on cell surface of both primary tumors and tumor cell lines, while it is not expressed by healthy nor stressed cells. The presence of B7–H6 on the surface of tumor cells makes them susceptible to NKp30-mediated killing by NK cells, while the binding of B7–H6 with NKp30c, the inhibitory isoform of NKp30, induce NK cells to produce IL-10. The ability of NKp30c isoform to inhibit NK cell functions has also been shown to affect the clinical outcome of tumors, since the expression of this splice variant on NK cells correlates with a poor prognosis of gastrointestinal sarcoma (Delahaye et al., 2011).

An additional reported ligand for both NKp30 and NKp46 is the membrane associated heparan sulfate proteoglycan (HSPG), which is expressed on the surface of various tumor cells (Bloushtain et al., 2004). However, whether this molecule is a true binding partner for NKp30 remains controversial (Warren et al., 2005). This notwithstanding, it has been reported that the presence of three basic amino acids within the exposed regions of the distal portion of the Ig domain of this NCR is essential for the binding of HSPG to NKp46 (Zilka et al., 2005; Ito et al., 2011). However, mutations of these three amino acids did not affect the binding of NKp46 to influenza virus HA indicating that NKp46, like NKp30, contains more than one binding site for the recognition of multiple ligands (Ito et al., 2011).

NKp44 has been shown to interact with the proliferating cell nuclear antigen (PCNA), which is commonly expressed by tumor cells and recruited to cell synapses with NK lymphocytes. In contrast to what has been observed for NKp46 and NKp30 ligands, the ligation of NKp44 with PCNA strongly inhibits both NK cell cytotoxicity and IFN-γ production (Rosental et al., 2011). The PCNA-mediated inhibition of NK cell function has been associated with the unusual presence of the inhibitory ITIM motif within the intracellular domain of NKp44 (Moretta et al., 2001).

## Expression and Function of NCRs on Immune Cells Other than NK Cells

**Figure 2:**
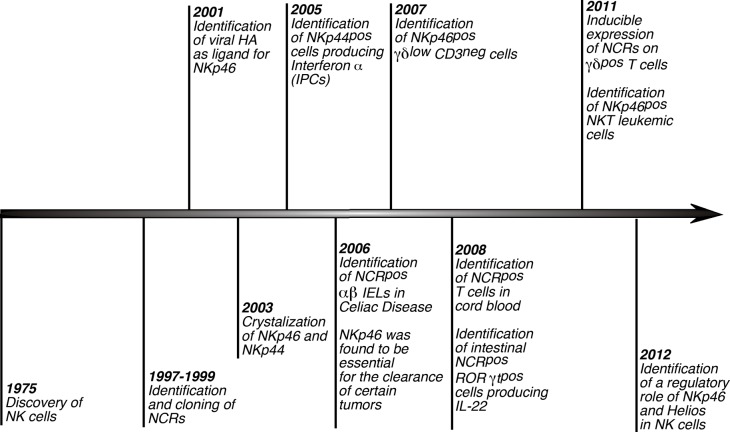
**History of NCRs**. Timeline scheme showing and referencing the distinctive hallmarks of NCRs since the discovery of NK cells. Of note, although these receptors were first described to play a key role in triggering NK cell cytotoxicity and cytokine secretions, several studies lately reported that NCRs are expressed also on non-NK immune cells.

Reviewing the recent literature, one can find many papers describing the expression of NCRs on non-NK or NK-like cell populations (Figure 2; Table 1). The expression of NKRs on cells other than NK cells is, however, not a new concept. Indeed, it is well known that that certain populations of T cells can express several inhibitory and activating NKRs such as KIRs, NKG2D, CD94/NKG2C (Hayday, 2000; Fauci et al., 2005; Parham, 2005; Borrego et al., 2006; Eagle and Trowsdale, 2007). Nonetheless, for several years the presence of a shared repertoire of NKRs between NK and non-NK cells did not include NCRs, whose expression had been originally described to be NK cell specific (Moretta et al., 2001). The recent and continued discoveries of NK-like immune cells and, more specifically, of new lymphoid subsets both in human peripheral blood and tissues has prompted some researchers to closely investigate whether NCRs could also be present and be functionally relevant on such cells.

**Table 1:** **Distinctive hallmarks of NCRs since the discovery of NK cells**.

Cell type	Species	NCRs	Induction mechanism	NCR functional correlates	Reference
Interferon producing cells (IPCs)	Human	NKp44	IL-3	Inhibition	Fuchs et al. (2005)
αβ T cell in small intestinal epithelium	Human	NKp44, NKp46	TCR and IL-15	Lysis	Meresse et al. (2006)
TCRγδ^low^ CD3^neg^	Mouse	NKp46	TCR	Unknown	Stewart et al. (2007)
Cord blood T cells	Human	NKp46, NKp44, NKp30	gc Cytokines	NKp30-dependent lysis and IFNγ production only	Tang et al. (2008)
RORγt^pos^/IL-22^pos^	Human, mouse	NKp46 (mouse), NKp44 (human)	Unknown	Unknown	Satoh-Takayama et al. (2008), Cella et al. (2009)
Expanded peripheral blood γδ T cells	Human	NKp46, NKp44, NKp30	TCR and γc cytokines	Lysis, chemokine production	Correia et al. (2011), Hudspeth et al. (2012)
NKT leukemia	Human, mouse	NKp46	TCR and IL-15	Unknown	Yu et al. (2011)

### Expression of NCRs on T lymphocytes

The original cluster of papers published in the late 1990s (Figure 2; Table 1) characterizing the phenotype and functions of human NCRs revealed a highly specific NK cell expression pattern of these receptors (Sivori et al., 1997; Pessino et al., 1998; Vitale et al., 1998; Cantoni et al., 1999; Pende et al., 1999; Moretta et al., 2001). One of the first reports showing the presence of NCRs on cell types other than NK lymphocytes demonstrated that *in vitro* expanded γδ T cell clones from the peripheral blood could express low levels of NKp44, but not NKp46 or NKp30, only after cell activation (Vitale et al., 1998). Later on, the expression of several NKRs, including NKp46, has been reported on human TCR αβ intestinal intraepithelial lymphocytes (IEL) (Jabri et al., 2000; Meresse et al., 2006). It was then hypothesized that the gut epithelium could favor the presence and/or the expansion of a population of CD3^pos^/TCR-αβ^pos^ cytotoxic NK-like lymphocytes expressing several NCRs and other NKRs during the course of intestinal inflammation in patients affected by celiac disease. It was subsequently disclosed that IL-15, a cytokine over expressed in celiac disease, could convert the aforementioned IELs into lymphokine-activated killers, which can lyse tumor cell targets in an NKG2D-dependent but TCR-independent manner (Meresse et al., 2004). Shortly after, the same group identified another NKR, NKG2C, as being up-regulated on cytolytic IELs during active celiac disease. Moreover, the induced expression of NKG2C was found to be associated not only with cytotoxicity, but also with cell proliferation, a function generally limited to the engagement of the TCR (Meresse et al., 2006). In particular, this study showed that NKG2C^pos^ cytolytic IELs also express a great repertoire of NK cell-related genes and receptors, including NKp46 and NKp44. Interestingly, it was shown that this subset of cells had a decreased expression of the TCR as compared to the NKG2C^neg^ counterpart. The two NCRs expressed were functional as shown in their ability to induce IFN-γ secretion and degranulation. These results suggested that certain inflammatory/activation conditions might favor the development/differentiation of non-NK cell populations (i.e., T cells), which can perform TCR-independent proliferation as well as NKR-mediated effector functions. Notwithstanding the autonomy of NCRs to drive cytotoxicity, the acquisition of an NK cell program in CD3^pos^ T cells from gut associated lymphoid tissues (GALT) appears to be dependent on previous TCR triggering as the ability to up-regulate NKp46 and NKp44 only occurs in effector T cells. Indeed, the expanded population of IELs expressing high levels of NCRs in celiac disease displays a highly restricted TCR repertoire indicative of a naturally occurring oligoclonal expansion (Meresse et al., 2006).

In line with the hypothesis that NCR induction on T cell surface follows a previous TCR-dependent cell activation, a population of “NK-like γδ T cells” expressing low amounts of the γδ TCR and containing high levels of intracellular CD3ε has been recently described in a mice (Stewart et al., 2007). These NK-like γδ T cells, which require the thymus and the recombinant activating gene 1 (RAG1) protein for their development, were also found to express several NKRs, such as NK1.1 and NKp46, although the cross-linking of this NCR does not induce the production of IFN-γ. In contrast, the production of IFN-γ was observed only when these cells were stimulated with IL-12 and IL-18. Moreover, despite the expression of several NKRs, NK-like γδ T cells are not able to lyse the NK cell-sensitive YAC-1 cell line. Future studies are required to exactly determine the function of NKp46 on these cells, but their ability to produce IFN-γ and to express various NKRs clearly indicates that NK-like γδ T cells may share with NK lymphocytes similar functional properties. The major current working hypothesis is that the acquisition of an NK cell program by these T cells is due to their chronic activation and several pieces of evidence support this theory. First, these cells express low levels of the γδ TCR, which is characteristic of chronic TCR engagement. Second, they display a memory T cell phenotype and have a high responsiveness to IL-15, a cytokine that favors survival of NK and memory CD8^pos^ T cells. Lastly, it has been shown that NKRs are highly expressed on CD8^pos^ T cells receiving chronic stimulation of the TCR, a phenomenon also confirmed by the finding that acute T cell activation in MCMV infected mice fails to up-regulate NKp46 on T cells (Walzer et al., 2007a). Taken together, these data suggest that NK-like γδ T cells are indeed T cells, which acquire an NK-like program following chronic activation that is capable of potentially driving NK-like functional outcomes independently from the engagement of TCR.

*In vitro* activation with IL-15 has been shown to induce the expression of NKp46 on human peripheral blood CD56^neg^/CD8^pos^ T cells as well as the surface levels of all three NCRs, with NKp30 being predominant, on T cells purified from cord blood (Tang et al., 2008; Correia et al., 2009). Among all the cytokines tested in this study, only IL-7 was unable to induce the expression of NKp30 on the surface of cord blood T cells. This is likely due to the fact that the *de novo* expression of these activating receptors is linked with cell division. Hence, the relative inability of IL-7 to induce cellular proliferation, as compared to that of IL-15 and IL-2, might explain the lack of a fully inducible expression of NCRs via IL-7. Of note, the *de novo* expression of NCRs is limited to those cord blood T cell subsets showing a cytolytic phenotype (either CD8^pos^ or CD56^pos^) and this phenomenon is equally distributed among TCR-αβ^pos^ and TCR-γδ^pos^ populations. Once again, these data suggest that certain T lymphocyte populations are able to acquire an NK-like phenotype following activation, which endows these cells with the ability to perform NK cell effector functions without prior antigen-specific sensitization. It is still unclear, however, what arms cord blood T cells with the ability to express NCRs after cytokine exposure, especially given the relative naivety of T cells purified from cord blood. In this regard, it has also been shown that the capacity to up-regulate NCRs is not a characteristic generally held by naïve cells as naïve T lymphocytes from the peripheral blood are not able to express NCRs *de novo* using the same cytokine stimuli (Tang et al., 2008). Hence, it is also possible that the unique microenvironment of the umbilical cord provides certain populations of T cells with the ability to up-regulate NCRs and this mechanism renders naïve T cells capable of responding immediately against potential dangers to the host, without the need for TCR-mediated antigen recognition.

Our group has recently characterized a population of human peripheral blood γδ T cells bearing the Vδ1 chain in the variable region of the TCR that can be induced to express *de novo* all three NCRs, with NKp30 showing the highest level of expression (Correia et al., 2011). Although circulating γδ T cells are NCR^neg^, we found that the surface levels of NKp30, NKp44, and NKp46 could be induced through the engagement of the TCR in synergy with cytokine stimulation (i.e., IL-2 or IL-15). These stimuli were able to induce selectively the expression of NCRs without affecting the surface levels of other NKRs such as DNAM-1, NKG2D, or 2B4. We observed that the *de novo* expression of NCRs (and of NKp30 in particular) correlates with cell proliferation, confirming once again that the NCR induction is associated with cell activation and division. In fact, our results indicate that NCR induction is dependent on both TCR stimulation and the presence of γc cytokines such as IL-15, which is present at high levels during the course of several inflammatory conditions (Carroll et al., 2008). Moreover, the hypothesis that the induced expression of NCRs is limited to certain populations of T cells carrying a particular TCR (Jabri et al., 2002; Meresse et al., 2006; Stewart et al., 2007; Yu et al., 2011) is also supported by our data showing that NCRs can only be induced on γδ T cells expressing the Vδ1 but not the Vδ2 TCR chain. We also showed that NCR^pos^ Vδ1 T cells have strong potential to be clinically relevant as tools in adoptive cell transfer (ACT) immunotherapies, as they are highly cytolytic against primary leukemia cells and against several tumor cell targets resistant to the lysis by Vγ9Vδ2 T cells. Moreover, we also demonstrated that these NCR^pos^ Vδ1 T cells are able to inhibit HIV viral replication, through the NKp30-mediated production of CCL3/MIP-1α, CCL4/MIP-1β, and CCL5/RANTES (Hudspeth et al., 2012). These three cc-chemokines are chemotactic proteins produced by cells of the immune system and are able to inhibit viral replication by binding CCR5, one of the primary co-receptors that HIV-1 utilizes for entry into CD4^pos^ T cells (Cocchi et al., 1995; Deng et al., 1996; Dragic et al., 1996; Feng et al., 1996). Collectively, these recent studies revealed that the induced expression of NCRs on γδ T cells from the peripheral blood endows these cells with important anti-viral and anti-tumor functions.

The importance of chronic activation given by soluble inflammatory factors on immune cells can be also assessed by using mouse models either over-expressing or lacking pro-inflammatory cytokines that are critical for immune cell stimulation. To this end, a transgenic mouse model over-expressing IL-15, a cytokine required for the homeostasis and proliferation of NK and CD8^pos^ T cells, was recently engineered (Yu et al., 2011). These mice display a massive expansion of NK, NKT, and CD8^pos^ T cells, but approximately 30% of them develop either a T cell large granular lymphocytes (T-LGL) or NK cell leukemia. The leukemic cells from T-LGL were CD1d restricted and, as such, this disease has also been defined as NKT cell leukemia. Surprisingly, these malignant NKT cells express high amounts of NKp46, despite the presence of only a small fraction of NKp46^pos^ T cells resident in bone marrow, spleen, thymus, and peripheral blood of wild type mice. ACT experiments clearly indicated that these cells do not originate from their NKp46^neg^ counterparts, but are the result of the expansion of those small subsets of NKp46^pos^ NKT cells present in blood and tissues of healthy mice. In line with these results, murine NKp46^pos^ T lymphocytes were remarkably more responsive to IL-15 stimulation compared to their NKp46^neg^ counterparts. Low frequencies of CD1d restricted NKp46 T lymphocytes have also been found in the peripheral blood from healthy human donors and, similar to what has been described in mice, they showed an increased responsiveness to IL-15 as compared to the NKp46^neg^ NKT cell subset. In patients diagnosed with T-LGL, both the surface expression of NKp46 receptor and transcript levels of NKp46 gene are much higher compared to those of healthy individuals and their CD1d restricted NKT cells are generally limited to invariant alpha and beta TCR chains (Yu et al., 2011). Moreover, it was also shown that NKp46^pos^ NKT cells are even more skewed toward certain Vβ chains, as compared to their NKp46^neg^ counterparts, which supports once again the hypothesis that the induced expression of NCRs is restricted to T cell subsets bearing only specific rearrangements of TCRs.

Finally, another line of investigation reported that the selective expression of NCRs on particular T cell subsets might represent a novel and useful biologic marker to track leukemia-transformed cells (Bensussan et al., 2011). In fact, it has been observed that circulating CD4^pos^ leukemia cells in patients affected by Sezary Syndrome express NKp46, but not NKp30 or NKp44. Sezary Syndrome is an aggressive form of CD4^pos^ T cell leukemia primarily localized in the skin, but with considerable leukemic cell invasions of the lymph nodes and the peripheral blood. In contrast to the well known activating function of NKp46, it was found this NCR could function as an inhibitor of the TCR-mediated proliferation of tumor cells through the hampering of CD3ζ chain phosphorylation.

The flexibility of T cells to acquire NK-like phenotype and functions has also been confirmed by a recent report showing that in the absence of the transcription factor B-cell lymphoma/leukemia 11B (Bcl11b), murine immature double negative (DN) thymocytes are “re-programmed” to express NK cell receptors, to produce IFN-γ and to efficiently lyse tumor cells in the absence of antigen-specific stimulus (Li et al., 2010). However, these induced T-to-natural killer (ITNK) cells were not entirely NK cells as they retained expression of CD3, T-bet, CD4, and CD8. Interestingly, this study also demonstrated the plasticity of T cells in the spleen, which can be re-programmed to ITNK in the absence of Bcl11b. These results indicate that T cells during development in the thymus as well as in the periphery are plastic in nature, although it is still unclear under what circumstances these NK-like T cells arise and it remains to be determined which T cells within the periphery are able to be re-programmed in the absence of Bcl11b.

### NCR expression on other cell types

Several studies in the last few years have described unique populations of tissue-associated CD3^neg^ innate lymphoid cells (ILCs) expressing NCRs but lacking classical NK cell functions, such as cytotoxicity and IFN-γ production. ILCs were originally identified in the murine intestine as NKp46^pos^ cells phenotypically similar to lymphoid tissue inducer (LTi) cells (Satoh-Takayama et al., 2008). LTi cells are a specialized subset of cells, which were originally described in mice as necessary for the proper growth of lymph nodes and requiring the transcription factor RORγt for their development (Colonna, 2009). Likewise, NKp46^pos^ LTi cells from murine intestine express RORγt and were also reported to produce IL-22 (Satoh-Takayama et al., 2008). IL-22 is a member of the IL-10 family of cytokines and is involved in the repair and maintenance of mucosal epithelial barriers (Colonna, 2009). Indeed, mice lacking intestinal IL-22^pos^/NKp46^pos^/RORγt^pos^ LTi cells were found to be more susceptible to *Citrobacter rodentium* bacterial infection (Satoh-Takayama et al., 2008). IL-22-producing cells expressing NKp46 and RORγt and with an LTi functional profile have been also found in human fetal mesenteric lymph nodes, tonsils, and the intestine as well as in murine cryptopatches (Cupedo et al., 2009; Luci et al., 2009; Sanos et al., 2009). It has also been reported that NKp44 is the major NCR expressed on a cell subset purified from human tonsils that produces IL-22 and expresses RORγt, which is functionally similar to NKp46^pos^/CD3^neg^ cells isolated from mouse Peyer’s patches (Cella et al., 2009).

It has also been found that cell surface density of the NCRs varies in RORγt^pos^ LTi cells isolated from different mucosal sites. While the tonsil and intestine contain IL-22^pos^/RORγt^pos^ LTi cells expressing high levels of NCRs, the cellular counterpart from both adult and fetal lymph nodes expresses very little amounts of NKp44 and NKp46 and do not produce IL-22, but instead contain transcripts for IL-17a (Hoorweg et al., 2012). These data indicate that RORγt^pos^ LTi cells represent a unique immune compartment regulating the homeostasis of mucosa associated lymphoid tissues (MALT) and that the expression of NCRs and production of cytokines by LTi cells vary according to their tissue distribution. Given their shared similarity with NK cells in terms of NCR expression and absence of surface molecules indicating terminal stages of cell differentiation, it was first hypothesized that LTi cells might be immature NK cell precursors. Among the experimental evidence supporting this thesis, there were the facts that (i) LTi cells lacking NKp46 could differentiate into cells expressing NKRs (including NKp46, NKp44, and NKp30), while maintaining LTi functions, and that (ii) the immature NK cell population purified from fetal lymph nodes has been found to contain transcripts for RORγt and IL-22 (Cupedo et al., 2009). However, despite the induction of NCRs on LTi cells following stimulation *in vitro*, the cross-linking of NKp46, NKp30, and NKp44 does not trigger cytotoxicity in LTi cells and the ligation of NKp46 is not required for the clearance of *C. rodentium* infection in mice (Cella et al., 2009; Luci et al., 2009; Satoh-Takayama et al., 2009). Only recently it has been clarified by several reports that, although similar in terms of NCR and RORγt expression, LTi cells and conventional NK lymphocytes belong to separate lineages and have distinct functional and transcriptional profiles both in mice and humans (Crellin et al., 2010; Satoh-Takayama et al., 2010; Vonarbourg et al., 2010; Narni-Mancinelli et al., 2012; Tomasello et al., 2012).

Finally, NKp44 was also found to be expressed on a small subset of IFN-α producing plasmacytoid dendritic cells (pDCs) isolated from human tonsils (Fuchs et al., 2005). Surprisingly, the presence of this NCR on pDCs is associated with the inhibition of TLR-7/9 mediated production of IFN-α, in contrast to the well-established activating function of NKp44 on NK cells (Moretta et al., 2001). The mechanism involved in the induction of NKp44 on tissue pDCs seems to be IL-3-dependent, as peripheral blood pDCs are induced to express NKp44 upon incubation with IL-3. It has been hypothesized that, due to their natural ability to produce IL-3 and their close proximity to pDCs in the tonsils, memory CD8^pos^ T cells play a key role in inducing the expression of NKp44^pos^ pDCs in human tonsils. Detectable surface levels of NKp44 have been reported on pDCs from lymph nodes, blood, and malignancies of patients affected by systemic lupus erythematosus (SLE) (Bonaccorsi et al., 2010). Although a possible association between SLE and IL-3 has never been fully investigated, the reported increased levels of IFN-α in the serum of SLE patients might be associated with dysfunctions of NKp44^pos^ pDCs (Gilliet et al., 2008).

## Functions of NCRs on Immune Cells Other than NK Cells

**Figure 3:**
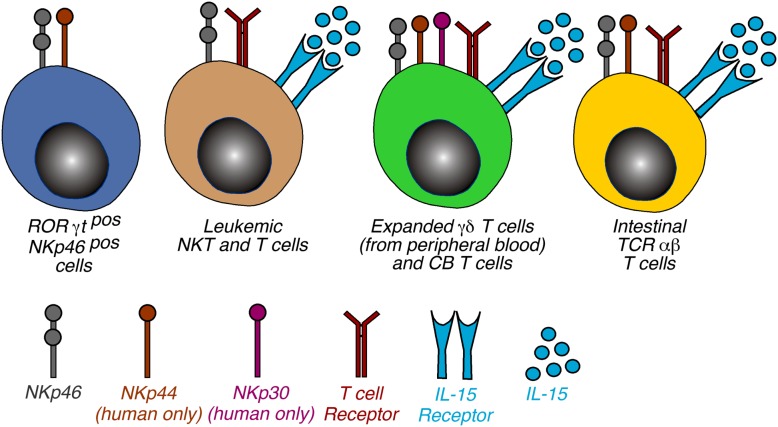
**Phenotypic distribution of NCRs on non-NK cells**. Schematic representation of non-NK cells expressing NKp46, NKp44, and NKp30. The 4 cell populations were categorized on the basis of cell type and NCR expressed and the receptor required to induce the NCR expression is also indicated. Of note, the mechanisms involving the triggering of TCR and γc cytokines are a common feature of NCR induction of NCRs on T lymphocytes. Abbreviations: CB, cord blood; IELs, intraepithelial lymphocytes.

While the role of the NCRs on some specialized lymphoid subsets such as ILCs or LTi cells is currently unknown or being debated, several pieces of evidence indicate that the expression of NCRs on certain T cell subsets is able to endow them with an NK-like program (Figure 3). The acquisition of an NK functional profile by NCR^pos^ T cells might be a consequence of a chronic activation of these cells, a phenomenon occurring in many human inflammatory conditions. Subsequently, the engagement of NCRs might bypass or substitute for the functions of the TCR in order to activate those intracellular signaling pathways needed for cell cytotoxicity, cytokine production, and cell proliferation. This appears to be a plausible scenario, since it has been shown that the ITAM-bearing molecules, like those associated with NCRs, can activate the Src tyrosine kinases, Lck, and Fyn, which are central in the initiation of TCR signaling pathways (Vivier et al., 2004).

The novel concept of NCRs representing an alternative pathway either substituting or synergizing with TCR signaling might open several new insights in regard to our knowledge of immune responses against tumors and pathogens. As previously stated, all experimental evidence collected so far has shown that very small frequencies of NCR^pos^ T cells are naturally resident in tissues and peripheral blood of both human and mice. Nevertheless, it has become evident that several activation stimuli, both TCR-dependent and independent, are able to establish pathways for the expansion of NCR^pos^ cells or for the *de novo* induction of NCRs on T cells with certain TCR rearrangements. One could speculate that these T cells with particular TCRs might be pre-programmed to express NCRs, an event that could occur in the thymus. Post-thymically, TCR-dependent mechanisms would pave the way for expression of NCRs, which could be induced under conditions of inflammation wherein IL-15 and IL-2 are in abundance.

On the other hand, aversion from the normal TCR-mediated mechanisms of tolerance could have also detrimental effects on the host. Indeed, stimulation of T cells through germ-line encoded receptors generally recognizing self-molecules and circumventing TCR stimulation could likely hinder T cell regulatory mechanisms and lead to unwanted and aberrant immune responses. As a physiological example of this scenario is represented by the expression of NCRs on IEL in celiac disease, an autoimmune disorder where the cytokine IL-15 can drive a pathologic expansion of TCR-αβ IELs with an NK cell-like phenotype. This mechanism has been proposed to contribute to the destruction of the intestinal epithelium in patients affected by this disease, although a complete elucidation of these pathogenic mechanisms has not yet been disclosed. In fact, it is still unknown whether NCR ligands are expressed on intestinal epithelial cells either constitutively or following inflammation, cell stress, or other stimuli. However, it has been shown that the expression of MHC-class-I chain-related molecules A and B (MICA/B) and non-classic HLA-E (i.e., the ligands of the C-type lectin NKG2D and NKG2C receptors, respectively) is indeed induced by inflammation and cellular stress, which also represent the main triggers for the expansion and activation of NCR^pos^ IELs (Groh et al., 1998; Meresse et al., 2004, 2006). Furthermore, NCR^pos^ T cells have the potential to become malignant under certain conditions (Yu et al., 2011), thus indicating that NCRs could serve as biological markers of immune dysregulation and/or potential leukemic transformation.

On the contrary however, the hypothesis that NCR^pos^ T cells might represent a bridge between innate and adaptive immunity is certainly fascinating as the NCR-mediated and the innate-like response by T cells against unwanted or dangerous cellular components could provide the host with an enhancement of the first line of defense against pathogens or tumor-transformed cells.

## Potential Therapeutic Approaches Using NCR^pos^ Non-NK Cells

The manipulation of NCRs, in particular on γδ T cells, might represent a promising new tool in ACT immunotherapies. First, the *de novo* expression of NCRs (NKp30 in particular) endows circulating Vδ1 T cells with potent cytolytic ability against primary leukemia cells resistant to the lysis exerted by the more abundant Vγ9Vδ2 cell subset (Correia et al., 2011), which have been the focus of essentially all γδ T cell-based cancer immunotherapy trials. This potential application is in line with the emerging paradigm of γδ T cells recognizing tumors via innate NK receptors rather than using the somatically rearranged TCR γδ (Gomes et al., 2010). This notwithstanding, the anti-tumor function of NKp30^pos^/Vδ1^pos^ cells has also a major, albeit indirect, contribution of the TCR. Indeed, the efficient induction of NKp30 expression on Vδ1^pos^ cells depends on TCR stimulation; in its absence, γc cytokines can only induce a very modest up-regulation of NKp30 expression. Thus, TCR signals are upstream of NKp30-mediated tumor cell recognition by NKp30^pos^/Vδ1^pos^ lymphocytes (Correia et al., 2011). Therefore, we have previously proposed a “two-step” model for human γδ T cells in which they differentiate and are activated like prototypic T cells following the engagement of TCR, but rely essentially on NK receptors (such as NCRs and NKG2D) for tumor cell recognition. This is consistent with the critical role described for NKG2D ligands in tumor surveillance by mouse γδ T cells (Girardi et al., 2001; Strid et al., 2008), and fits the general concept of NK receptors being the key molecular recognition determinants of “oncogenic stress” (Raulet and Guerra, 2009). The role of NCRs in the potential ACT might be even more relevant for eradicating several hematological tumors that lack ligands to NKG2D (Hayday, 2000). From a clinical perspective, the establishment of a clinical protocol inducing the expression of NCRs on Vδ1^pos^ cells *ex* *vivo* and injecting large numbers of these cells into patients affected by tumors is feasible. Indeed, we previously reported that NKp30^pos^/Vδ1^pos^ cells could be efficiently expanded from patients affected by B-cell chronic lymphocytic leukemia. Upon reinfusion, the activation status of the cells could be potentially maintained via administration of low doses of IL-2, which appears to be sufficient to sustain NKp30 expression (Correia et al., 2011).

The finding that the NKp30 engagement on *in vitro* activated Vδ1 T cells is able to suppress HIV-1 replication through the NKp30-induced production of CCL3/MIP-1α, CCL4/MIP-1β, and CCL5/RANTES also reveals new and important insights for understanding the role and for manipulating the functions of γδ T cells during HIV-1 disease. Indeed, HIV-1 infection is characterized by a great expansion of Vδ1 T cells (Autran et al., 1989; Hinz et al., 1994; Boullier et al., 1995; Poles et al., 2003) and by chronic inflammation (Appay and Sauce, 2008). In such conditions, activated and expanded Vδ1 T cells producing cc-chemokines might contribute to the control of viral replication. This phenomenon is likely to be even more relevant in mucosal tissues, given that intestinal and cervical mucosae, where Vδ1 T cells are naturally resident in high frequencies (Hayday, 2000), are two important gates of HIV-1 entry. In this context, NKp30^pos^ Vδ1 T cell-mediated control of viral replication might play an important role in restraining the establishment of viral reservoirs and in limiting the levels of mucosal and systemic inflammation generated by the pathologic translocation of microflora in the lamina propria and mesenteric lymph nodes following the massive depletion of mucosal CD4^pos^ T cells in acute and highly viremic stages of HIV-1 infection (Brenchley et al., 2006). In line with what was recently demonstrated for Vγ9Vδ2 T cells in SIV-infected primates (Ali et al., 2009), the establishment of novel protocols either inducing NKp30 expression on expanded Vδ1 T cells *in vivo* or adoptively transferring *in vitro* differentiated NKp30^pos^ Vδ1 T cells may be of great therapeutic value in HIV/AIDS.

## Concluding Remarks

The expression of NCRs on a variety of cell populations other than NK cells is a relatively new field of research that has changed the classic paradigm of these receptors being NK cell specific and opened several new avenues for research and clinical applications. With regard to T cells, the induction of NCR expression has been linked to their acquisition of an NK cell-like functional profile, which may be the result of chronic activation or simply a previous antigen recognition via the TCR. Furthermore, the expression of NCRs has also been described on populations of tumor-transformed T cells, which has further validated the hypothesis that the presence of NCRs follows a prolonged cellular activation or stress even on pathologic T lymphocytes. In this context, the use of NCRs as a tool to identify T cells potentially undergoing leukemic transformation as well as those that have already undergone transformation presents exciting opportunities for cancer research, given the lack of markers associated with certain T cell leukemias. Moreover, the use of NCRs as biomarkers of T cells that in certain inflammatory and autoimmune conditions have lost immune tolerance might represent a valuable tool for the diagnosis of autoimmune disorders. Lastly, *in vitro* manipulation to induce NCR expression can arm T cells with potent anti-tumor and anti-viral activity, thus offering a potentially powerful therapeutic tool for ACT immunotherapies.

## Conflict of Interest Statement

The authors declare that the research was conducted in the absence of any commercial or financial relationships that could be construed as a potential conflict of interest.
